# Supplementary material for: Direct observation of phase transitions in truncated tetrahedral microparticles under quasi-2D confinement
Source: Nat Commun. 2024 Mar 25;15:1954. doi: 10.1038/s41467-024-46230-x (PMC10963743; doi:10.1038/s41467-024-46230-x)
Supplement: Supplementary file 1 — Supplementary Information [file 41467_2024_46230_MOESM1_ESM.pdf]

Supplementary Information

Direct observation of phase transitions in truncated tetrahedral microparticles under quasi-2D confinement

Authors: David Doan[1]†, John Kulikowski[1]†, X. Wendy Gu[1]*

Affiliations: [1]Department of Mechanical Engineering, Stanford University; Stanford, California, 94305, USA.
* Corresponding author. Email: xwgu@stanford.edu
† These authors contributed equally to this work

This PDF file includes:

Supplementary Figures 1 to 5
Supplementary References

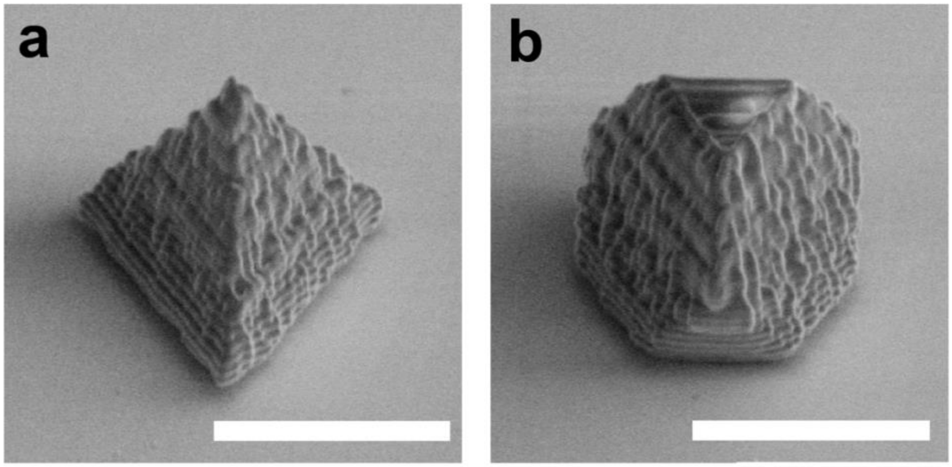

Supplementary Fig. 1. **Additional tetrahedral particles made using two-photon lithography.** SEM image and 3D model of **a** tetrahedron with a side length of 7 μm and **b** a non-standard truncated tetrahedron with side lengths of 3.3 μm (on the triangular face) and 2.8 μm (on the hexagonal face edges). Scale bars are 5 μm.

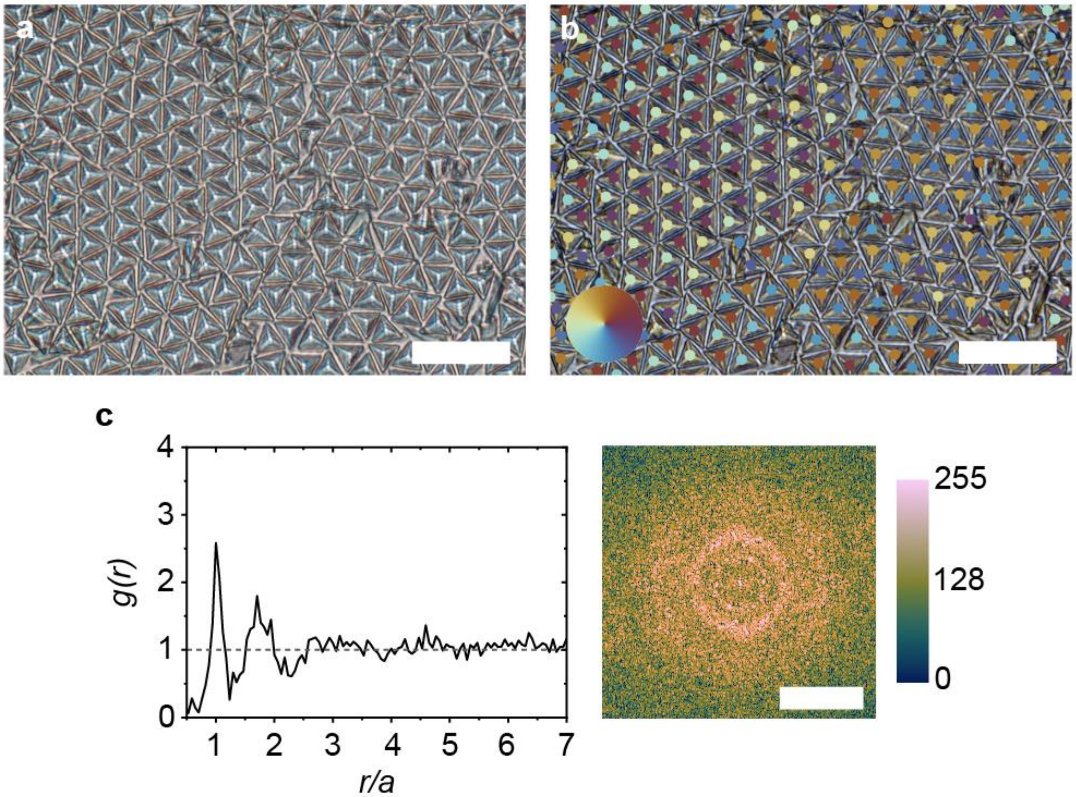

Supplementary Fig. 2. **Regular tetrahedrons self-assembled at a ≈10 degree tilt angle.**

a Optical image of assembled 2D array of regular tetrahedra with triangular faces aligned with the substrate. Particles generally have three neighboring particles that are close-packed in the plane of the substrate with 3-fold symmetry. Grains of ≈40 μm in size are separated by point or line defects that lead to misorientation between grains. Scale bar is 20 μm. **b** Bond orientational order parameter of the particles represented as different colors. Color wheel is shown in the bottom left. Neighboring particles within the grain have opposite colors on the color wheel because they are rotated by 60 degrees to form the repeating pattern. Scale bar is 20 μm. **c** Pair distribution function, $g(r)$, and Fourier transform of the image in **a**. The $g(r)$ and Fourier transform show a strong first peak at 4.3 μm, followed by a weaker second peak, which is indicative of a 3-fold liquid crystal phase. However, there is no long-range order, as the peaks decay beyond the second peak. Scale bar is 0.5 μm^{-1}. Color bar ranges from 0 to 255 (grayscale).

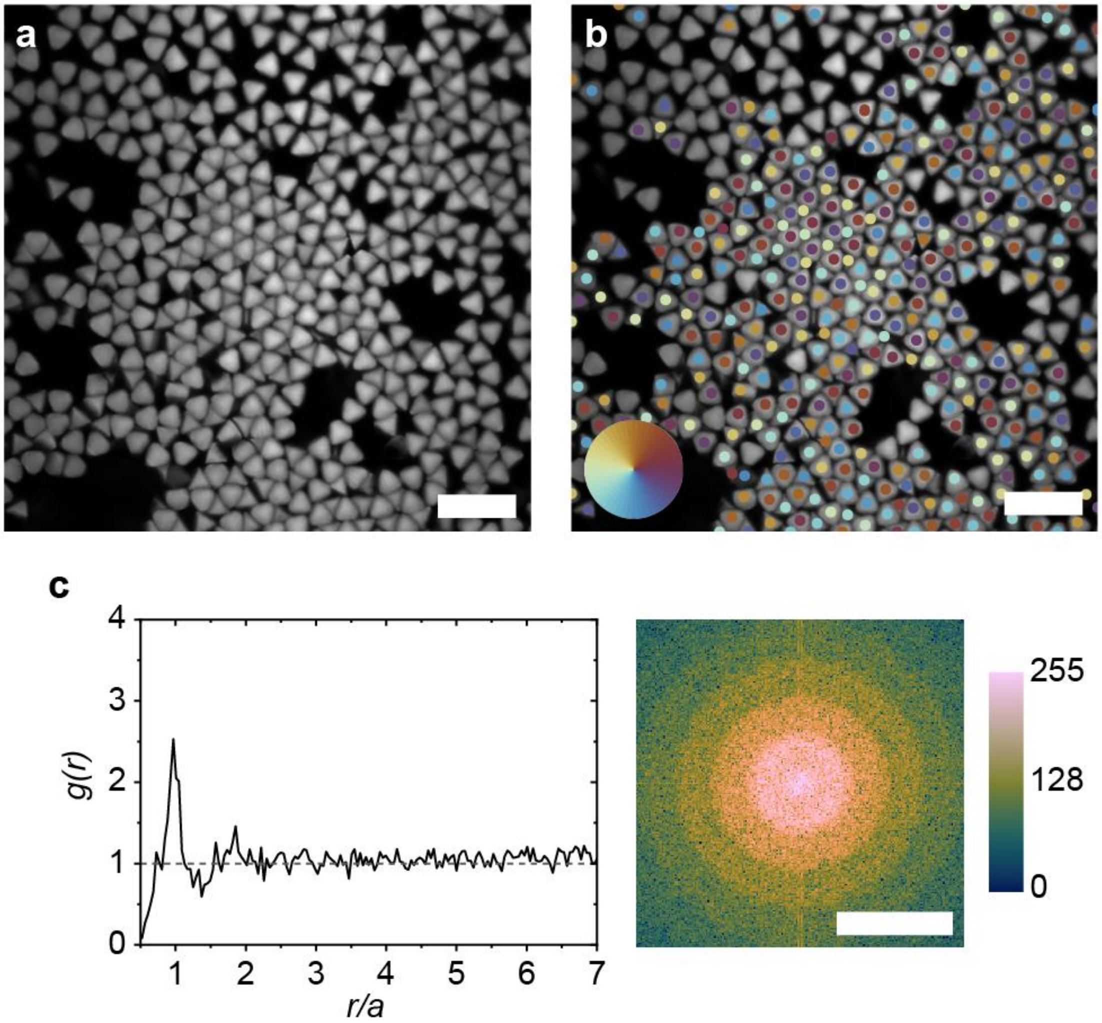

Supplementary Fig. 3. **Assembly of truncated tetrahedrons (*t* = 7/10) at a ≈ 15 degree tilt.**

a Confocal image of truncated tetrahedra (*t* = 7/10). This assembly is generally disordered, with small grains (≈10 μm) towards the center of the image that are similar to the quasi-diamond structure seen in the ATTs. Smaller grains form than with ATT particles because the *t* = 7/10 truncated tetrahedra do not stack together as well. Scale bar is 20 μm. **b** Bond orientational order parameter of the particles represented as different colors. Neighboring particles within the grain have opposite colors on the color wheel because they are rotated by 60 degrees to form the repeating pattern. Scale bar is 20 μm. **c** Pair distribution function, *g*(*r*), and Fourier transform of the image in **a**. The *g*(*r*) and Fourier transform show a first peak at ≈6.3 μm with no other peaks following, indicating a liquid or disordered phase. Scale bar is 0.5 μm^{-1}. Color bar ranges from 0 to 255 (grayscale).

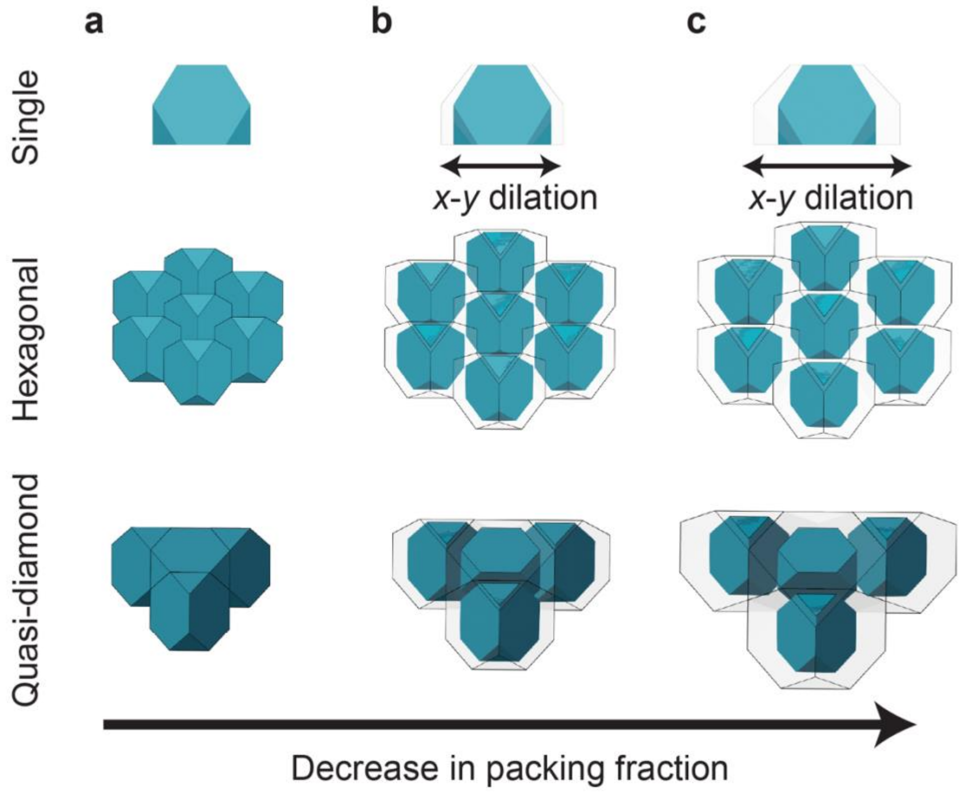

Supplementary Fig. 4. **Single-cell occupancy model**. Model of an ATT (teal) in a single, hexagonal, and quasi-diamond structure, with a self-similar (transparent) cell constructed around it at **a** the highest packing fraction, **b** lower packing fraction, and **c** lowest packing fraction. The free volume is available volume allowable for the ATT to move within its self-similar cell.

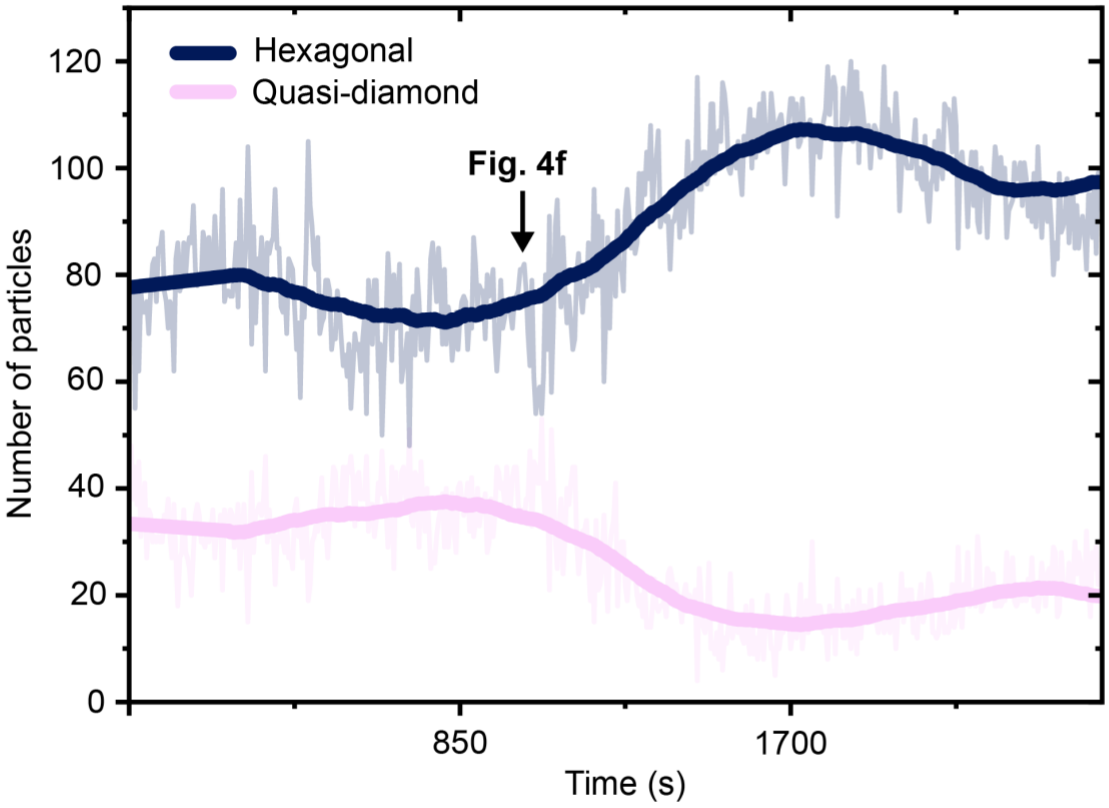

Supplementary Fig. 5. **Phase transition of hexagonal to quasi-diamond.**

Number of particles in each phase as a function of time for the phase transition from the hexagonal phase (navy blue) to the quasi-diamond phase (pink). Arrow indicates the first particle rotation and corresponds to Fig. 4f. The phase transition rate is modelled using the Avrami growth model[1,2]. This kinetic solid-solid phase growth model predicts that the transition will occur according to the following equation, $Y = 1 - e^{-Kt^n}$, where Y is the fraction of quasi-diamond to hexagonal phase particles, K is a rate constant, t is time, and n is the growth exponent. Fitting this curve after the initial out-of-plane rotation of the first hexagonal phase particle shows a growth exponent of $n \approx 1.8$, which is indicative of disk-like or rod-like growth (2D growth) after the initial particle flip.

Supplementary References

1. Avrami, M. Kinetics of Phase Change. I General Theory. *J. Chem. Phys.* **7**, 1103–1112 (1939).
2. Avrami, M. Kinetics of Phase Change. II Transformation-Time Relations for Random Distribution of Nuclei. *J. Chem. Phys.* **8**, 212–224 (1940).
