# Supplementary material for: Direct observation of phase transitions in truncated tetrahedral microparticles under quasi-2D confinement
Source: Nat Commun. 2024 Mar 25;15:1954. doi: 10.1038/s41467-024-46230-x (PMC10963743; doi:10.1038/s41467-024-46230-x)
Supplement: Supplementary file 3 — Description of Additional Supplementary Files [file 41467_2024_46230_MOESM3_ESM.pdf]

Description of Additional Supplementary Files

File Name: Supplementary Movie 1
Description: In-situ optical video of the Brownian motion of Archimedean truncated tetrahedrons under a 0-degree tilt.

File Name: Supplementary Movie 2
Description: Hard particle Monte Carlo simulation of transition of Archimedean truncated tetrahedrons from liquid to hexagonal to quasi-diamond.

File Name: Supplementary Movie 3
Description: In-situ optical video of vacancy mediated phase transition of one hexagonal grain to quasi-diamond.

File Name: Supplementary Movie 4
Description: In-situ optical video of anti-phase boundary mediated phase transition of hexagonal to quasi-diamond.

File Name: Supplementary Movie 5
Description: In-situ optical video of a phase transition with multiple particles flipping events that transform a grain from hexagonal to quasi-diamond.
